# Disparity in the Burden of Caring for Older Persons between Families Living in Housing Estates and Traditional Communities in Thailand

**DOI:** 10.3390/ejihpe14060100

**Published:** 2024-05-28

**Authors:** Nadila Mulati, Myo Nyein Aung, Saiyud Moolphate, Thin Nyein Nyein Aung, Yuka Koyanagi, Siripen Supakankunti, Motoyuki Yuasa

**Affiliations:** 1 Department of Global Health Research, Graduate School of Medicine, Juntendo University, Tokyo 113-8421, Japan; m.nadila.vp@juntendo.ac.jp (N.M.); moyuasa@juntendo.ac.jp (M.Y.); 2 Faculty of International Liberal Arts, Juntendo University, Tokyo 113-8421, Japan; 3 Advanced Research Institute for Health Sciences, Juntendo University, Tokyo 113-8421, Japan; 4 Department of Public Health, Faculty of Science and Technology, Chiang Mai Rajabhat University, Chiang Mai 50300, Thailand; saiyud_m@cmru.ac.th; 5 Department of Family Medicine, Faculty of Medicine, Chiang Mai University, Chiang Mai 50200, Thailand; drthinnyeinaung@gmail.com; 6 Global Health and Chronic Conditions Research Group, Chiang Mai University, Chiang Mai 50200, Thailand; 7 Faculty of Health Sciences, Tokyo Ariake University of Medical and Health Sciences, Tokyo 135-0063, Japan; y-koyanagi@juntendo.ac.jp; 8 Centre of Excellence for Health Economics, Faculty of Economics, Chulalongkorn University, Bangkok 10330, Thailand; siripen.s@chula.ac.th

**Keywords:** population aging, family caregiver, long-term care system, residential environment, caregiver burden, health disparity

## Abstract

Thailand’s rapid population aging and reliance on family-based long-term care requires research into disparities in family caregiver burden. Since the type of residence matters to the caregiving outcome, this research aimed to examine the difference in caregiver burden between residents of private housing estates and traditional village communities. This cross-sectional study was conducted with 1276 family caregivers of community-dwelling Thai older adults, in Chiang Mai province, Thailand. The caregiver burden was examined using the Caregiver Burden Inventory (CBI), and the care recipients’ dependency status was examined using Barthel’s Activity of Daily Living (ADL). Descriptive analysis, multivariate analysis of variance test, and multiple logistic regression analysis were performed. Family caregivers living in a traditional village community were 1.607 times more likely to experience emotional burden (adj. OR 1.607, 95% CI: 1.049, 2.462) and 2.743 times more likely to experience overall caregiver burden (adj. OR: 1.163, 95% CI: 1.163, 6.471) compared to those in the private housing estate group. Our findings showed significant differences in caregiver burden based on residential area, contributing with insights to evidence-based policies, interventions, and programs to minimize disparities and promote family caregivers’ health and well-being.

## 1. Introduction

Thailand is experiencing a fast pace of population aging and is expected to rank among the top ten countries globally in terms of its older population [1,2]. The country has successfully implemented universal health coverage (UHC), but due to the high cost, it does not cover the cost of any institutional long-term care [3]. The long-term care (LTC) program is not institution-based. It focuses on community-based and home care services, and most Thai older adults still rely on family members for support [3,4].

Like most developing countries, in Thailand, taking care of loved ones and parents is deeply ingrained in Thai tradition and cultural values. It serves as a means of expressing gratitude to those who have supported and assisted them throughout their lives [5]. According to a national survey, one in ten older Thai people has a caregiver, and the demand for caregivers tends to increase with age [2]. The percentage of older people in Thailand who have caregivers is 4% among the young-old (60 to 69 years), 11.4% among the middle-old (70 to 79 years), and 35.6% among the old-old adults (80 years or older) in 2021, and nearly 60% of the caregivers are the child of the older person [2]. 

The aging population, coupled with a declining birth rate and a shrinking working-age population, has led to a projected decline in Thailand’s potential support ratio; from 5.4 working-age adults per elderly person in 2020, this ratio is expected to decrease to 1.9 by the year 2050. This significant shift highlights a substantial reduction in the number of working-age individuals available to provide support and care for older adults [3]. Therefore, exploring the determinants of various caregiving outcomes is an essential topic for designing evidence-based programs, and interventions to promote the health and well-being of the family caregivers, as they remain indispensable in this context.

Caregiving burden, which is one of the common caregiving outcomes, can be defined as “the level of multifaceted strain perceived by the caregiver from caring for a family member and/or loved one over time” [6]. Family caregivers experience physical, psychological, emotional, social, and financial burdens due to providing care [7,8]. Scholars examined the wide range of factors correlated with the family caregiver burden, for instance, family caregiver characteristics, socio-economic status, the context, duration, and intensity of caregiving, the timing of entry into the caregiving role, relationship quality with care recipients, and the social and cultural context, and little is known about the effect of the physical environment on the family caregiver burden [9,10,11,12]. 

In Thailand, traditionally, people live in the *Muban* or village communities. Gradually, people have begun occupying *Mubanchatsan,* or gated housing estate, developed by private housing developers since the 1960s [13]. The structural factors, including the globalization of the economy, and the privatization of security, as well as the subjective factors (for instance, the desire for privacy, status, social homogeneity, the fear of crime, and investment potential) contributed to the rise in private housing estate communities [14,15]. While the political, social, and spatial effects of living in private housing estate communities are well explored, there is a gap in the literature examining its effect on health [13,15,16,17,18]. Residential environments, whether private housing estates or traditional village communities, offer distinct social and structural contexts that can influence the caregiving experience. Exploring caregiver burden across diverse residential types allows for a more comprehensive understanding of the complex interplay between socio-environmental factors and caregiving burden. 

Therefore, this study aims to examine the differences in family caregivers’ burdens residing in traditional village communities and private housing estate communities. 

## 2. Materials and Methods

### 2.1. Study Design and Setting

This is a cross-sectional study aiming to examine the caregiver burden of family caregivers residing in different neighborhoods. The study setting was Maehia City, Chiang Mai Province, Northern Thailand. The older population (age ≥ 60) consisted of more than 20% of the population in the study site [19].

### 2.2. Data Source

The data of this study originated from the baseline data of “Community-Integrated Intermediary Care (CIIC) Service Model to Enhance Family-Based, Long-Term Care for Older People: A Cluster Randomized Controlled Trial in Thailand: TCTR20190412004” [20]. This two-arm parallel intervention study consisted of 6 intervention clusters and 6 control clusters in Chiang Mai province, Thailand, that aimed to reduce the caregiver burden and promote the health of Thai community-dwelling older adults by designing a novel aging care model consisting of care capacity building for family caregivers, respite care services in the community and an exercise program for community-dwelling older adults. The baseline survey applied led to data collection and this study analyzed the response of the caregivers.

STATA version 11SE (Stata Corporation, College Station, TX, USA) was utilized for sample and power estimations. The estimated sample consisted of 1500 participants in each arm to determine the size difference of 0.5 units with a standard deviation (SD) of 4 between the two arms [21]. The precision levels applied are a *p*-value of 0.05 with a 95% confidence interval. The sample size was inflated by up to 20% for design effect of cluster randomized design application, and compensation of potential non-responses and drop-outs during the recruitment and study [21].

A total of 1276 family caregivers and their care recipients were randomly selected both from the intervention arm and the control arm. The inclusion criteria were as follows: care recipients over 60 years of age and their family caregivers (both male and female) who are taking care of the older adults at home and residing in the study site for at least one year with written informed consent. The exclusion criteria were persons who had cognitive impairment and were unable to understand informed consent, or who did not consent. Data were collected from August to December 2019 by a well-trained public health research assistant following the research protocol [20].

### 2.3. Measurement

The instruments used in this study are validated instruments commonly used in research on aging and long-term care. They have been translated into the Thai language and validated in previous studies and programs in Thailand.

#### 2.3.1. The Caregiver Burden

The caregiver burden inventory (CBI) is a multidimensional measure used to assess the burden of family caregivers [22]. It is an internally validated, 5-point Likert scale which encompasses five dimensions: time-dependent burden (5 items), developmental burden (5 items), physical burden (4 items), social burden (5 items), and emotional burden (5 items). The time-dependent burden pertains to the limitations placed on the caregiver’s time. The developmental burden reflects caregivers’ feelings of being out of sync with their peers in terms of personal development. The physical burden encompasses caregivers’ experiences of chronic fatigue and physical health deterioration. Social burden encompasses caregivers’ feelings of role conflict. Lastly, the emotional burden captures caregivers’ negative emotions toward the individuals they care for [22]. The total score of CBI equals the sum of the 24-item scale. The application of the total score alone cannot portray the unique difference in the burden the caregiver experienced. Therefore, by using both the five dimensions and the total burden score, we can gain a clearer understanding of the caregivers’ experiences. To achieve this, we categorized the responses as follows: “Never” was coded as 0 = no burden, while “Rarely”, “Sometimes”, “Quite Frequently”, and “Nearly Always” were combined and coded as 1 = rarely to always burden across the five dimensions (time dependence, developmental burden, physical, social, and emotional burden). Moreover, the total score of CBI was categorized into two groups; (1) the total score lower than 24 group (coded as 0); and (2) the total score equal to or more than 24 group (coded as 1), indicating high burden.

#### 2.3.2. Independent Variable: Residential Type

The participants were asked if they resided in private housing estate communities (coded as 0) or traditional village communities (coded as 1).

#### 2.3.3. Caregiver and Care Recipient Characteristics

The caregiver and care recipients’ characteristics included socio-demographic information, relationships, health conditions, and lifestyle factors.

Caregivers’ socio-demographic information included age, sex (coded male as 0, female as 1), and marital status (which was categorized as a not married group (including single, separated, divorced, and widowed, (coded as 0), and married group (coded as 1)), educational background (no formal education (coded as 0), primary school completed (coded as 1), secondary school and above completed (coded as 2, including secondary school, the vocational school completed, bachelor degree or above completed)), and working status (not working coded as 0, working coded as 1). Monthly income was asked, and the answer ranged from no income to THB 300,000; since it was scattered, we categorized it using the median value, ≤10,000 group (coded as 0), and >10,000 group (coded as 1). Whether they were the main income supporter of the family or not was also asked (yes coded as 0, no coded as 1). Moreover, the relationship of the caregiver with their care recipients was categorized into spouse (coded as 0), children or son/daughters-in-law (coded as 1), sibling (coded as 2), and others (relative, maid, etc., coded as 3). Willingness to send the care recipients to short-term aging care facility were asked (yes = 0, no = 1). Lifestyle factors included smoking habits, alcohol consumption, and exercise habits. Smoking and drinking alcohol were categorized into yes (including occasional and currently smoking or drinking, coded as 0), and no groups (including never smoked or drank or quit, coded as 1). Exercise behavior includes participants who do not exercise (coded as 0), who exercise but not regularly (coded as 1), and who exercise regularly (coded as 2). The underlying diseases of the study participants included hypertension and diabetes (no coded as 0, yes as 1) were included as the indicator of the health state of the family caregivers.

Care recipients’ socio-demographic information consisted of age (completed years), and sex (male coded as 0, female as 1). Variables regarding health condition were whether they had diabetes mellitus or hypertension (yes = 0, no = 1), and the dependency status. The dependency status was measured using the Barthel Index of Activity of Daily Living (ADL). It is a standard measure used and validated in Thailand to assess the dependency status of older adults. It examines the ability to perform ten daily basic activities, consisting of bathing, feeding, dressing, toilet use, transfer, indoor mobility, grooming, stairs, bowel control, and bladder control. The total possible scores range from 0 (maximal disability) to 20 (maximal independence). We categorized functional ability as mildly dependent to independent (total ADL score ≥12, coded as 0), moderately dependent (total ADL score = 5~11, coded as 1) and severely dependent groups (total ADL score = 0~4, coded as 2) [23]. Finally, we asked their willingness to use the respite care center provided by the municipality (yes = 0, no = 1), to explore the cultural norms in this matter.

### 2.4. Statistical Analysis

The data were analyzed using the STATA version SE17 (Stata Corp 4905, Lakeway Drive, College Station, TX 77845, USA). Descriptive analysis was performed to understand the study participants’ characteristics. Frequency and percentage were used for categorical variables, and mean, standard deviation (SD) for continuous variables.

To assess whether there were statistically significant differences in caregiver burden dimensions between two residential types, the Multivariate Analysis of Variance (MANOVA) test for the total score of time dependence, developmental, physical, emotional, and social burden dimensions, and the Wilcoxon sign-rank test for the total caregiver burden were conducted.

Then, binary logistic regression analysis was conducted to identify an association between dependent variables (time dependence, developmental burden, physical, social, and emotional burden), categorized as (0 = no burden, 1 = rarely to almost always burden), total CBI burden (coded 0 as a score <24 no burden and ≥24 high burden) and independent variable (residential type) as odds ratio and 95% confidence interval.

Since the binary logistic regression analysis results showed statistical significance on the emotional burden and the CBI total burden, we conducted multiple logistic regression analyses to identify the adjusted odds ratio and 95% confidence interval as a measure of association between the independent variables, type of the houses, and dependent variables, which are emotional burden categorized into two groups (0 = no burden, 1 = rarely to always burden), and total caregiver burden (coded 0 as a score < 24 no burden and ≥24 high burden). A *p*-value of less than 0.2 in binary analysis and conceptually related factors were included in the model. The dependent variables (caregiver emotional burden and overall burden) were adjusted for age, sex, marital and education status, work status of the family caregiver, and age and sex of care recipients, separately. Statistical significance was defined as *p* < 0.05 with a 95% confidence interval (CI). The fit of each multiple logistic regression model was checked applying Hosmer–Lemeshow goodness-of-fit test.

## 3. Results

### 3.1. The Characteristics of the Study Participants

The characteristics of the family caregivers and their care recipients are shown in Table 1. A total of 1276 family caregivers of community-dwelling older adults participated in this study, with a mean age of 54.87 (SD = 13.94) years. Most of the participants were female (62%), married (71.5%), and working (71.6%) at the time the study was conducted. Among them, 81.6% were residents of traditional village communities, while 18.4% resided in private housing estates. Notably, residents of traditional village communities were older, with a mean age of 55.73 (SD = 13.91) years, compared to residents of private housing estates, who had a mean age of 51.05 (SD = 13.44) years, and this difference was statistically significant (*p* < 0.001). Moreover, the family caregivers of the private housing estate community had a higher educational background, with 80.4% of them completing secondary school or above; this percentage was 59.8% for the family caregivers of the traditional village community (*p* < 0.001). As for the relationship of the family caregivers and their care recipients, spouses were most common in the traditional village community, accounting for 49.2%. In contrast, in the private housing estate group, children and son/daughter-in-law were the most prevalent caregivers, representing 50.9%. More than half (57.4%) of the study participants’ monthly income was > THB 10,000, and they were the main income supporter of the family (52.8%). The willingness of family caregivers in the traditional village community (30.5%) to send older adults to the municipality-provided short-term care center was significantly higher compared to those in the private housing estate group (6.0%).

In terms of lifestyle factors, most study participants did not smoke (91.8%) or consume alcohol (73.7%). Only 12.1% of family caregivers reported regular exercise, with a slightly higher percentage observed among the traditional village community group (12.5%) compared to the housing estate group (10.2%). Regarding health status, family caregivers in the traditional village community group had a higher percentage of individuals with diabetes (11.9%) and hypertension (27.7%), whereas these figures were 4.3% and 21.7%, respectively, for caregivers of the private housing estate group (Table 1).

The mean age of the care recipients was 69.34 (SD = 8.26) years, with most of them being female (57.5%), and able to independently perform basic daily activities (96.8%) by themselves (ADL mean score of 19.15 (SD = 2.81)). Care recipients of the original village community were slightly older, 69.59 (SD = 8.42) years, than those in the private housing estate, 68.21 (SD = 7.42) years (Table 1). Moreover, the prevalence of hypertension and diabetes among the care recipients was also higher in the traditional village community group (48.9% and 20.3%, respectively) compared to that in the private housing estate group (46.0% and 11.5%, respectively). When asked about their willingness to use short-term aging care facilities in their municipality, a higher proportion of care recipients in the traditional village community (32.1%) answered yes compared to those in the private housing estate community group (12.1%). This difference was statistically significant (*p* < 0.001).

### 3.2. The Difference in the Burden Experienced by the Family Caregivers in Two Residential Types

**Figure 1:**
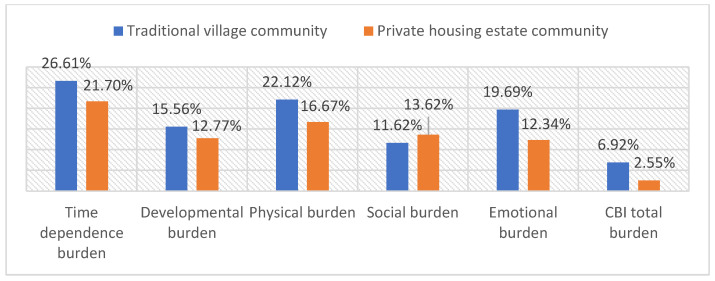
Percentage of family caregivers who experienced high burden (score > 24) by residential type in Chiang Mai, Thailand (N = 1276).

The burden of the family caregiver is multidimensional. Family caregivers residing in traditional village communities experience a greater burden across the dimensions of time dependence (26.61%), developmental (15.56%), physical (22.12%), and emotional (19.69%) burden compared to those in private housing estate communities. The social burden was slightly higher in the private housing estate community group (13.62%). Overall, the high total burden was experienced more among the family caregivers of the traditional village community group (6.92%) compared to those in the private housing estate group (Figure 1).

Moreover, the type of residential community significantly influences the overall caregiver burden experienced by family caregivers. Table 2 shows the MANOVA test results, which indicate the statistically significant differences in caregiver burden dimensions (time dependence, developmental, physical, emotional, and social burden) between the two types of residential areas (private housing estate and original village community). Additionally, adjusting for the age and sex of both caregivers and care recipients confirms that the observed differences are not solely due to demographic factors but are related to the type of residential area (Table 2).

Additionally, the total caregiver burden was also statistically significantly different among family caregivers residing in the original village community and those in the private housing estate community (*p* < 0.05) (Table 2).

### 3.3. Association of Residential Type and Caregiver Burden

Context matters to the caregiving outcome. The binary logistic regression analysis showed that there is a statistically significant association between residential type and the likelihood of experiencing overall caregiver burden and emotional burden (*p* < 0.05) (Table 3).

The emotional burden of family caregivers often refers to caregivers’ negative feelings towards their care recipients. After adjusting for the confounders, family caregivers of the original community group were 1.607 times more likely to experience emotional burden (adj. OR 1.607, 95% CI: 1.049, 2.462) compared to those in the private housing estate group. Moreover, regardless of the socio-economic status of the family caregiver, and age and sex of the care recipients, they were 2.743 times more likely to experience overall caregiver burden (adj. OR: 1.163, 95% CI: 1.163, 6.471) (Table 3).

## 4. Discussion

The caregiving experience is intricately linked to its contextual surroundings [24]. While the literature extensively examines the social, cultural, and policy contexts, there is a noticeable gap in the exploration of the neighborhood context [25,26,27,28]. Residential settings can vary widely, from private housing estates to traditional village communities, each presenting unique challenges and resources for the family caregivers.

Our study results showed a statistically significant difference in the burden experienced by the family caregivers residing in different residential contexts (Table 2). Although most of the care recipients in this study were able to perform daily activities independently (Table 1), caregivers still experience burden at various levels and in different aspects (Table 2). Moreover, even accounting for potential confounding factors, caregivers residing in original village communities showed a significantly higher likelihood of experiencing a high caregiver burden compared to those in private housing estates (Table 3). This disparity highlights the complex interplay between residential environments and caregiver experiences, suggesting that factors essential to traditional village communities may contribute to increased caregiver burden. Socio-economic factors contribute to the informal caregiving outcome [29]. In this study, these factors could include the socio-demographic disparity among the family caregivers of traditional village communities, including older age, lower educational background, and higher prevalence of non-communicable disease (Table 1).

On the one hand, Bronfenbrenner’s Theory of Human Ecology emphasizes reciprocal interaction between individuals and their environment [30]. The development and functioning of an individual are the result of the interaction between its micro (immediate surroundings), meso (neighborhood and institutional level and the physical environment), exo (the economic, political, and educational systems), macro (cultural and political ideologies) and chrono (socio-historical conditions and patterns of events and transitions over a life course) systems [30]. The caregiving outcome and experience are also the product of the interaction of all the above systems. On the other hand, according to Pearlin et al.s’ stress process model, the caregiver burden is the subjective primary stressor, and it is influenced by the caregivers’ socio-economic characteristics. The burden then directly and indirectly affects the physical and psychological health outcome of caregiving. And these can be mediated by the coping and social support of caregivers [31].

A supportive physical and social environment has a positive impact on caregivers [32,33]. The physical characteristics of the private housing estates in Thailand include security guards and walls, club services and amenities (vending machines, convenience store, laundromat, swimming pool, gym, and garden), and the economic status of its residents varied from low- to high-income class [13,17]. Studies showed that the burden experienced by family caregivers can be modified by access to leisure facilities [34]. Participation in leisure activities, for example, hobbies, and socializing, is a form of self-care that is important for the well-being of family caregivers by contributing to the coping capacity, and stress relief [35]. Private housing estate communities are equipped with a range of facilities and amenities that are not available in traditional village communities, and this can be one of the factors contributing to the lower burden experienced by family caregivers in private housing estate groups [36].

In this study, the family caregivers and the care recipients of the traditional village community were older (Table 1). The burden increases with age [7,37]. The age factor can contribute to the higher burden experienced by family caregivers of the original village community [38]. Marital status can also serve as a predictor of caregiver burden, with married caregivers often facing a higher likelihood of experiencing an elevated burden compared to those who are unmarried [39]. In this study, the proportion of married family caregivers was higher in the traditional village community group (Table 1). Drawing from role theory, which posits that individuals juggle various societal roles, the absence of adequate support and time for caregiving can lead to role overload and conflict [40]. Married caregivers may find themselves navigating the dual roles of spouse and caregiver, potentially increasing the burden they experience. The correlation between educational background and caregiver burden is varied. However, a lower educational background may pose a risk of experiencing a higher burden [41]. Some studies suggest that caregivers with higher education levels are also susceptible to experiencing higher burden [42]. The family caregivers in the private housing estate community had higher educational backgrounds, which might contribute to their coping capacity. The health state of the caregiver and care recipients is associated with the caregiving outcome as well [43,44]. The prevalence of diabetes and hypertension among the family caregivers and care recipients was higher in the traditional community group (Table 1). Managing their health while taking care of others can contribute to the higher burden. The above-mentioned socio-demographic factors have proven to be determinants of caregiver burden in the same study context in another study [7,12,45].

Another important factor is the cultural practice of hiring domestic workers (housekeepers, helpers, maids) in Thailand [46]. Although this study did not specifically investigate the role of domestic workers, it is common for affluent households to engage with their services. This is particularly prevalent in private housing estate communities. Moreover, caregivers of the original village community were more willing to use the short-term stay facility in the municipality (Table 1). Studies showed that the high burden of family caregivers and lower social resources contribute to the increased willingness to use respite care facilities [47,48]. These phenomena further demonstrate the disparities in accessible resources. Such disparities may potentially widen health inequalities among caregivers across various residential types, especially in countries with limited access to universally available formal long-term care systems. Therefore, innovative ideas for strengthening the existing family-based long-term care are necessary, like the “Community-Integrated Intermediary Care (CIIC) Service Model” to minimize the disparity of burden, and promote equity, fostering an environment where all caregivers receive equitable support, regardless of their residential setting [21].

The multidimensionality of the burden was observed among the study participants. High burden in time dependence, developmental, physical, emotional, and social burdens were observed among the family caregivers of traditional village community participants (Table 2, Figure 1), which highlighted the necessity for the support of family caregivers in multiple aspects. Common programs and interventions to reduce the caregiver burden include general education, support groups, behavior therapy, psychotherapy and counseling, and respite care [8]. Van Houtven CH et al. developed an organizational framework for caregiver interventions, which highlights major components including caregiver and care recipient characteristics (demographics, health, economics, insurance, relationship, and cultural norms), caregiving activities (skills, knowledge, psychological coping, support seeking, and time spent caregiving), caregiver outcomes (psychological and physical health, healthcare usage, and economic status), and care recipient outcomes (disease management, psychological and physical health, healthcare usage, respite care, and economic status) [49].

The strength of the study is the huge sample size and measurement of caregivers’ burden by applying an internationally validated instrument. Sampling bias was prevented by inflating the sample size to compensate possible non-response by 20%. Possible confounders such as age and education were adjusted in multiple logistic regression analysis. However, this study is not without limitations. Firstly, due to the nature of the cross-sectional study design, a causal relationship between residential type and caregiver burden should be interpreted by the reader carefully. And the use of a binary variable for household income may not fully capture the economic realities of caregivers. Secondly, further research should explore factors such as available neighborhood resources and the social networks of older adults to better understand factors contributing to the disparity of burden experienced by the original village community and private housing estate communities. Moreover, as the prevalence of frailty and dementia increases with aging, future research aiming to assess the caregiver outcome and presence of frailty, cognitive impairment, and behavioral problems of the care recipients in different types of housing is also necessary. However, given the rapid demographic change of the aging population and the lack of a sustainable formal long-term care system, our study results provide valuable insights into the underlying mechanisms driving caregiver burden disparities and informs targeted interventions to reduce the burden on family caregivers.

## 5. Conclusions

Minimizing disparities in family caregiver burden is crucial for ensuring equitable access to care resources and promoting the well-being of both caregivers and older adults. Thailand, a country with universal health coverage for more than twenty years, is still preparing to establish long-term care services and a system to face the challenge of a forthcoming super-aging society very soon. The results of our study showed that caregivers of traditional village communities were more likely to experience a higher caregiver burden. When there are significant differences in the level of burden experienced by caregivers in different residential settings, these can intensify existing inequalities in health outcomes and access to support services. Caregivers’ burnt-out and job losses challenge the sustainability of family-based long-term care. Moreover, older adults receiving care may also experience varying levels of quality and continuity of care depending on their caregivers’ level of burden. The results of this study contribute insights to make evidence-based policies, interventions, and programs to lessen the disparities of burden experienced by the family caregivers, building a more equal and inclusive caregiving environment.

It is urgent to systematically introduce caregiver support through holistic approaches such as respite care, care competency training, financial support, social support, legislation for caregiver leave, psychological support for family caregivers and health promotion.

## Figures and Tables

**Table 1:** Socio-economics, lifestyle, and health status of family caregivers and their care recipients in Chiang Mai, Thailand (N = 1276).

	Residential Type			
	Traditional Village Community	Private Housing Estate Community	Total	*p*-Value
Total Participants	1041 (81.6%)	235 (18.4%)	1276 (100.0%)	
Socio-demographics				
Age, Mean (SD)	55.73 (13.91)	51.05 (13.44)	54.87 (13.94)	<0.001
Sex				0.082
Male	384 (36.9%)	101 (43.0%)	485 (38.0%)	
Female	657 (63.1%)	134 (57.0%)	791 (62.0%)	
Marital status				<0.001
Not married	270 (25.9%)	94 (40.0%)	364 (28.5%)	
Married	771 (74.1%)	141 (60.0%)	912 (71.5%)	
Educational background				<0.001
No formal education	21 (2.0%)	3 (1.3%)	24 (1.9%)	
Primary school completed	398 (38.2%)	43 (18.3%)	441 (34.6%)	
Secondary school and above completed	622 (59.8%)	189 (80.4%)	811 (63.6%)	
Working status				0.831
Not working	294 (28.2%)	68 (28.9%)	362 (28.4%)	
Working	747 (71.8%)	167 (71.1%)	914 (71.6%)	
**Income**				0.882
≤10,000	408 (42.5%)	87 (43.1%)	495 (42.6%)	
>10,000	552 (57.5%)	115 (56.9%)	667 (57.4%)	
Main income supporter for the family?				0.900
Yes	549 (52.7%)	125 (53.2%)	674 (52.8%)	
No	492 (47.3%)	110 (46.8%)	602 (47.2%)	
Relationship with their care recipients				<0.001
Spouse	296 (49.2%)	75 (33.2%)	371 (44.9%)	
Children, son/ daughter in law	247 (41.1%)	115 (50.9%)	362 (43.8%)	
Siblings	34 (5.7%)	16 (7.1%)	50 (6.0%)	
Others (relatives, grandchildren)	24 (4.0%)	20 (8.9%)	44 (5.3%)	
Willingness to send the care recipients to short-term aging care facility				
Yes	194 (30.5%)	14 (6.0%)	208 (24.0%)	<0.001
No	441 (69.5%)	218 (94.0%)	659 (76.0%)	
Lifestyle factor				
Exercise behavior				0.480
Do not exercise	166 (15.9%)	43 (18.3%)	209 (16.4%)	
Exercise, but not regularly	745 (71.6%)	168 (71.5%)	913 (71.6%)	
Regularly exercise	130 (12.5%)	24 (10.2%)	154 (12.1%)	
Smoking				0.038
Yes	77 (7.4%)	27 (11.5%)	104 (8.2%)	
No	964 (92.6%)	208 (88.5%)	1172 (91.8%)	
Alcohol consumption				0.003
Yes	256 (24.6%)	80 (34.0%)	336 (26.3%)	
No	785 (75.4%)	155 (66.0%)	940 (73.7%)	
Health status				
Hypertension				0.062
Yes	288 (27.7%)	51 (21.7%)	339 (26.6%)	
No	753 (72.3%)	184 (78.3%)	937 (73.4%)	
Diabetes				0.001
Yes	124 (11.9%)	10 (4.3%)	134 (10.5%)	
No	917 (88.1%)	225 (95.7%)	1142 (89.5%)	
Care recipients’ characteristics				
Age, Mean (SD)	69.59 (8.42)	68.21 (7.42)	69.34 (8.26)	0.031
Sex				0.680
Male	445 (42.7%)	97 (41.3%)	542 (42.5%)	
Female	596 (57.2%)	138 (58.7%)	734 (57.5%)	
Diabetes				0.002
yes	211 (20.3%)	27 (11.5%)	238 (18.7%)	
no	830 (79.7%)	208 (88.5%)	1038 (81.3%)	
Hypertension				0.416
Yes	509 (48.9%)	108 (46.0%)	617 (48.4%)	
No	532 (51.1%)	127 (54.0%)	659 (51.6%)	
Dependency status, Mean (SD)	19.17 (2.81)	19.09 (2.79)	19.15 (2.81)	0.112
Dependency status in three categories				
Mildly dependent to independent	227 (96.6%)	1008 (96.8%)	1235 (96.8%)	0.902
Moderately dependent	5 (2.1%)	18 (1.7%)	23 (1.8%)	
Severely dependent	3 (1.3%)	15 (1.4%)	18 (1.4%)	
Willingness to stay at short-term aging care facility				<0.001
Yes	204 (32.1%)	28 (12.1%)	232 (26.8%)	
No	431 (67.9%)	204 (87.9%)	635 (73.2%)	

Note: SD: standard deviation, *p*-value calculated from Chi-square test and Mann–Whitney *U* test.

**Table 2:** Difference in the caregiver burden experienced by the family caregivers of traditional village and private housing estate communities (N = 1276).

	Traditional Village Community	Private Housing Estate Community	Total	*p*-Value
	Mean (SD)	Mean (SD)	Mean (SD)	
Time dependence burden	1.413 (3.430)	1.362 (3.779)	1.404 (3.495)	* <0.0001
Developmental burden	0.681 (2.036)	0.434 (1.317)	0.636 (1.926)	
Physical burden	1.112 (2.909)	0.686 (2.035)	1.034 (2.773)	
Emotional burden	0.812 (2.164)	0.545 (1.814)	0.763 (2.106)	
Social burden	0.505 (1.645)	0.532 (1.639)	0.510 (1.643)	
Total burden	4.523 (9.791)	3.559 (8.206)	4.345 (9.524)	^#^ 0.015

Note: * MANOVA (adjusted for the age and sex of family caregivers and care recipients), ^#^ *p*-value from Mann–Whitney *U* test.

**Table 3:** Association between housing type and family caregiver burden (N = 1276).

		Private Housing Estate Community	Traditional Village Community
Time dependence burden	OR (95% CI)	1	1.308 (0.932, 1.836)
Developmental burden	OR (95% CI)	1	1.259 (0.829, 1.913)
Physical burden	OR (95% CI)	1	1.420 (0.977, 2.060)
Social burden	OR (95% CI)	1	0.834 (0.549, 1.267)
Emotional burden	OR (95% CI)	1	1.742 (1.147, 2.645) *
	Adj. OR (95% CI)	1	1.607 (1.049, 2.462) *
Total burden	OR (95% CI)	1	2.836 (1.218, 6.603) *
	Adj. OR (95% CI)	1	2.743 (1.163, 6.471) *

Note: Adj. OR: Adjusted Odds Ratio. 95%CI: 95% confidential interval. The multiple logistic regression models for each outcome were adjusted for age, sex, educational background, marital status, the working status of the family caregiver, and age, and sex of the care recipients. * *p*-value < 0.05.

## Data Availability

The data presented in this study are available on request from the corresponding author due to privacy reason.
